# RNA editing in cardiovascular health and disease

**DOI:** 10.1038/s42003-026-09680-1

**Published:** 2026-02-11

**Authors:** Xiaoxin Huang, Charles Solomon, David G. McVey, Shu Ye

**Affiliations:** 1 Division of Basic Medicine, Shantou University Medical College, Shantou, China; 2 Division of Cardiovascular Sciences, University of Leicester, Leicester, UK; 3 Leicester British Heart Foundation Centre of Research Excellence, University of Leicester, Leicester, UK; 4 The First Affiliated Hospital of Shantou University Medical College, Shantou, China; 5 Cardiovascular-Metabolic Disease Translational Research Programme, National University of Singapore, Singapore, Singapore

**Keywords:** Gene regulation, Gene expression

## Abstract

Post-transcriptional RNA modifications can alter RNA structure, stability, localization, and function. Adenosine-to-inosine (A-to-I) RNA editing is a post-transcriptional modification that converts adenosine nucleotides in RNA to inosine nucleotides, catalyzed by adenosine-deaminase-acting-on-RNA (ADAR) enzymes. Recent studies have shown that A-to-I RNA editing is required for cardiovascular development and homeostasis whilst aberrant RNA editing plays a role in cardiovascular diseases. This article provides an overview of A-to-I RNA editing events that have been implicated in cardiovascular biology and disease. It also discusses harnessing RNA editing for cardiovascular disease biomarker development and engineering RNA editing for cardiovascular disease treatment.

## Abstract (web summary)

This review summarizes reported findings showing that adenosine-to-inosine RNA editing is important for cardiovascular development and homeostasis, while aberrant editing is associated with cardiovascular diseases.

## Introduction

RNA editing is a type of post-transcriptional modification that leads to an alteration of the RNA sequence. RNA editing was first discovered in *Trypanosoma brucei* in 1986^1^. Subsequently, in 1989, it was also found to exist in mammals including humans^2^.

**Fig. 1:**
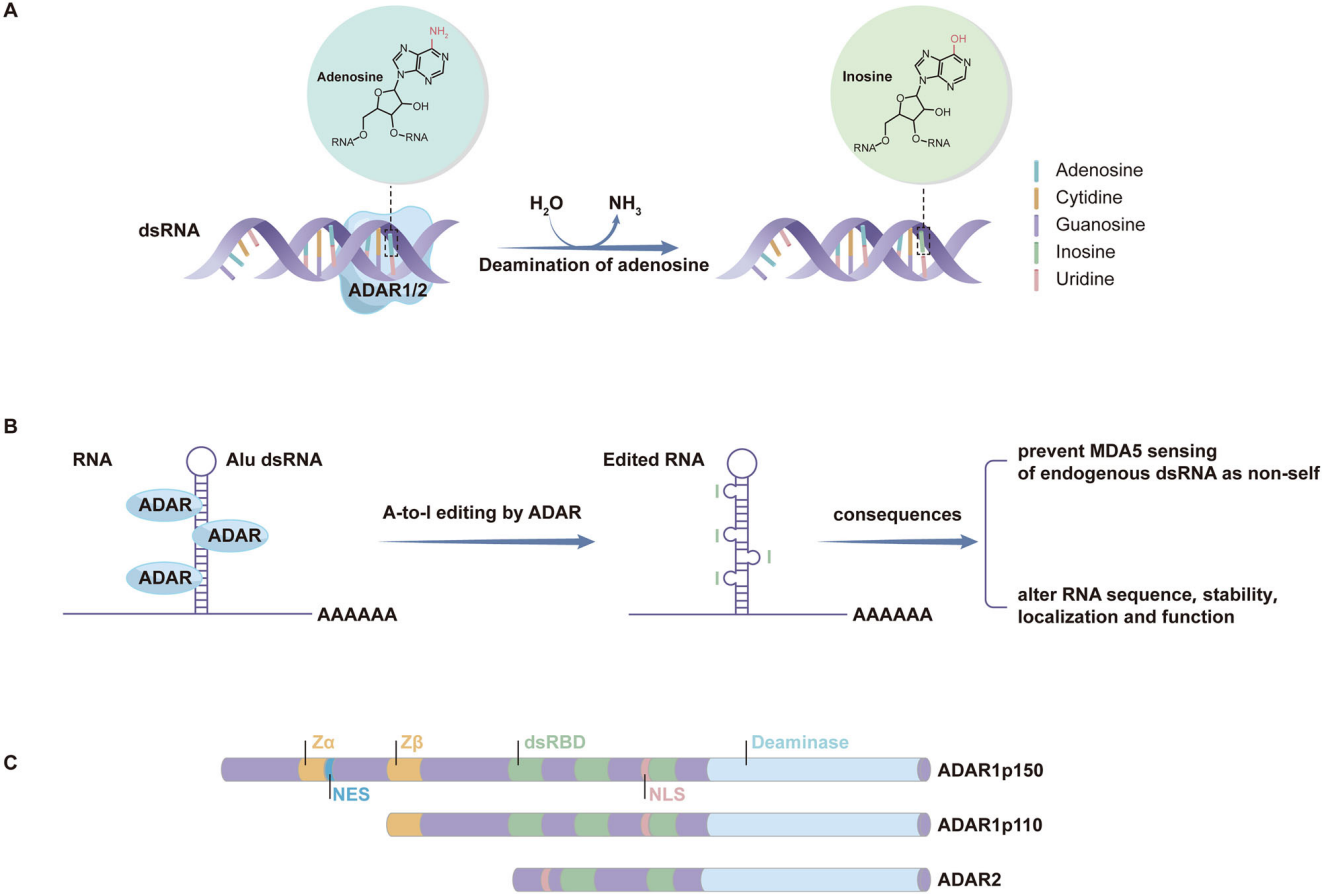
Diagram of adenosine (A)-to-inosine (I) RNA editing. **A** ADAR (adenosine deaminase acting on RNA) enzymes recognize specific sites on double-stranded RNA (dsRNA) and deaminate its adenosine, thereby converting it to inosine. **B** Editing of Alu double-stranded RNAs (dsRNAs) and its consequences. RNA editing mostly occurs on regions of inverted Alu repeats in the introns and untranslated regions of the genome. These Alu repeats form intramolecular RNA duplexes (Alu dsRNAs) which serve as substrates for ADAR enzymes for A-to-I RNA editing. Reported consequences of A-to-I RNA editing include preventing MD5 sensing of endogenous dsRNA as non-self, and altering the sequence, stability, localization, and function of RNA molecules. **C** The various domains of the two ADAR1 isoforms (ADAR1p150 and ADAR1p110) and ADAR2. All these enzymes share a conserved catalytic deaminase domain (indicated in blue) and two or three double-stranded RNA-binding domains (dsRBD, indicated in green) that determine substrate specificity. The two isoforms of ADAR1 differ in the number of Z-nucleic acid-binding domains (Zα and Zβ; indicated in yellow), which are the main distinguishing features between them. NES, nuclear export signal; NLS, nuclear localization signal.

The most common type of RNA editing is adenosine-to-inosine (A-to-I) editing which is catalyzed by ADAR (adenosine deaminase acting on RNA) enzymes. These enzymes deaminate adenosines in double-stranded RNA and thereby convert them to inosines (Fig. 1)^3^. Inosines are recognized as guanosines by the RNA splicing and translation machineries^3^.

A-to-I RNA editing has long been considered to be a self-protective mechanism to prevent false activation of the innate immune system by endogenous double-stranded RNAs^4^. Indeed, defects in RNA editing have been linked to some autoimmune diseases^5^. Additionally, aberrant RNA-editing has been associated with various cancers and neurological disorders^5^.

In recent years, a breadth of studies has discovered that RNA editing is highly abundant in cardiovascular tissue^6^ and plays an important role in cardiovascular health and disease. Here we aim to provide an overview of these findings, with a focus on A-to-I editing because the other types of RNA editing have been less studied in this subject area.

Additionally, we will discuss the possibility of leveraging RNA editing for cardiovascular disease biomarker development and engineering RNA editing as a novel approach for cardiovascular disease treatment.

Apart from A-to-I editing, there are several other types of RNA editing. To date, there is no direct evidence implicating these other types of RNA editing in cardiovascular development or disease. Therefore, this review will not discuss them in detail but only briefly describe them in the immediately following paragraphs.

The second most common type of RNA editing is cytosine to uridine (C-to-U) editing driven by members of the APOBEC (apolipoprotein B mRNA editing enzyme catalytic polypeptide-like) family, that catalyze the deamination of cytosines (C) to uridines (U). U is recognized as a T^7^. The best-known example of this type is the C-to-U editing at nucleotide position 6666 of the *APOB* mRNA, which leads to a premature stop codon, resulting in a truncated protein known as apoB48, while the unedited *APOB* mRNA produces the full-length protein named apoB100^7^. This process plays an important role in lipid metabolism, as apoB48 is synthesized in the intestine to transport dietary lipids, while apoB100 is made in the liver to transport endogenously synthesized fats^8,9^. Although high apoB level is a cardiovascular risk factor^10^, there is hitherto no reported evidence implicating a direct role of *APOB* mRNA editing in cardiovascular disease pathogenesis.

It has been reported that APOBEC3A cytidine deaminase induces C-to-U editing in monocytes and macrophages^11^. This could potentially be relevant to cardiovascular diseases, as monocytes and macrophages are key players in the pathogenesis of atherosclerosis which underlies coronary heart disease and contributes to several other cardiovascular diseases^12^.

In addition to A-to-I and C-to-U editing, 10 other types of RNA editing have been reported^13^, however, they are very rare and it is unknown if they influence any biological processes or disease development.

## A-to-I RNA editing enzymes in cardiovascular development, health and disease

Three members of the ADAR family have been identified in vertebrates including humans, namely ADAR1, ADAR2 and ADAR3^14–16^. ADAR1 and ADAR2 are responsible for A-to-I editing via their ability to deaminate adenosines to inosines in double-stranded RNA (Fig. 1)^17,18^. ADAR1 is the primary editor of RNA from repetitive elements, whilst ADAR2 is the primary editor of coding region-derived RNA^19^. ADAR1 and ADAR2 are thought to have almost non-overlapping target specificities and non-redundant functions, including their respective effects in cardiovascular disease^20–22^. ADAR3 is catalytically inactive^16^ and acts as a negative regulator of RNA editing by competing with ADAR1 or ADAR2 for binding to RNA substrates^23,24^.

Studies of cell and animal models with manipulation of these genes have indicated that ADAR1 and ADAR2, but not ADAR3, are crucial for cardiovascular development and homeostasis.

### ADAR1

ADAR1 is ubiquitously expressed in most tissues. ADAR1 has two isoforms, a 110 kDa isoform (p110) and a 150 kDa isoform (p150), which are transcribed from different promoters of the *ADAR1* gene^25^. The p110 isoform is constitutively expressed, whilst p150 is induced by interferon signals^25^. ADAR1 p110 is localized in the nucleus, while ADAR1 p150 is present in both the cytoplasm and nucleus^25^. Both isoforms of ADAR1 have A-to-I RNA editing activity ^25^. Additionally, it has been reported that this enzyme can exert editing-independent effects on gene expression, RNA splicing, and microRNA processing ^26^.

Studies have indicated that ADAR1 is required for embryonic development, as global knockout of the *Adar1* gene in mice is embryonically lethal and results in developmental defects in multiple organs, including the heart. Wang et al. reported that *Adar1* null mice developed normally up to embryonic day 10.5 but subsequently had increased cellular apoptosis in the heart and other tissues, and died in late-stage gestation^27^. As ADAR1 may have other functions apart from RNA editing, Liddicoat et al. investigated specifically the role of RNA editing by Adar1 in a study of mice with an A-to-I editing-deficient knock-in mutation of *Adar1*^28^. They found that such mice died at embryonic day 13.5^28^, suggesting an RNA editing-specific effect.

Studies of cell-type specific knockout of the *Adar1* gene have further demonstrated that it plays important roles in the cardiovascular system. Moore et al. found that mice with cardiomyocyte-specific deletion of *Adar1* died embryonically, with evidence suggesting reduced cardiomyocyte proliferation and increased cardiomyocyte apoptosis^29^. Garcia-Gonzalez et al. showed that inactivation of Adar1 enzymatic activity in murine cardiomyocytes caused autoinflammatory myocarditis which progressed into dilated cardiomyopathy and heart failure at 6 months of age, with activated interferon pathways^30^.

Guo et al. reported that 75% of mice with endothelial cell-specific knockout of *Adar1* died within 3 weeks after birth, with multiple organ abnormalities^31^. They showed that vascular endothelial cells isolated from such mice had reduced proliferation rates and decreased migration ability and tube formation^31^. These changes were associated with innate immune activation and elevated expression of interferon-stimulated genes^31^.

Furthermore, Cai et al. showed that tamoxifen-induced vascular smooth muscle cell (VSMC)-specific knockout of *Adar1* led to lethality in adult mice 14 days after induction, with multiple deleterious vascular effects, including extensive hemorrhaging, vascular damage in multiple organs, and destruction of arterial structural integrity characterized by the detachment of elastin laminae from VSMCs and disruption of the elastin and fibrillin-1 interaction^32^. Deletion of *Adar1* also resulted in pronounced VSMC apoptosis and mitochondrial dysfunction^32^.

Taken together, the findings from the abovementioned studies indicate that ADAR1, likely through its action in A-to-I RNA editing, is indispensable in cardiovascular development and plays an important role in maintaining the homeostasis of various cell types of the cardiovascular system.

Furthermore, a recent study by Weldy et al. reveals that ADAR1 has a protective effect against atherosclerosis, the pathology underlying coronary artery disease (CAD). The authors showed that in a mouse model, VSMC-specific knockout of ADAR1 increased the size and calcification of atherosclerotic lesions via activation of Mda5 (melanoma differentiation-associated protein 5), a key intracellular sensor of double-stranded RNA in innate immune response.

### ADAR2

ADAR2 is highly expressed in the brain and at lower levels in other tissues, including the heart and arteries^15^. ADAR2 has two isoforms: ADAR2a and ADAR2b, which are produced by alternative splicing^33^. Compared with ADAR2a, ADAR2b has 40 more amino acid due to the insertion of an Alu cassette^33^. The two isoforms act on the same set of RNA molecules but ADAR2a has a higher catalytic activity than ADAR2b^33^.

It has been reported that global *Adar2* knockout mice are prone to seizures and die between day 0 and 20 after birth, suggesting that Adar2 is required for survival^34^. Recent studies have indicated that ADAR2 plays an important role in response to ischemic injury to the heart. Wu et al. showed that AAV9 (adeno-associated virus serotype 9)-mediated cardiac-specific *Adar2* overexpression attenuated ischemia-induced apoptosis of cardiomyocytes and reduced the size of acute myocardial infarction in a mouse model^21^. In another study, Gatsiou et al. showed that Adar2 is required for the expression of the interleukin-6 (IL6) receptor in vascular endothelial cells and is therefore involved in IL6-mediated response to tissue injury ^22^. This study also demonstrated that whilst the lL-6 signaling pathway promoted immune cell trafficking to sites of sterile inflammation (including ischemic heart), this was affected by *Adar*2 knockout in vascular endothelial cells^22^.

### ADAR3

ADAR3 expression is limited to the brain^16^. It has been shown that Adar3 knockout mice are largely normal, but they have increased levels of anxiety and deficits in memory^35^. There is no report of a direct effect of ADAR3 in the cardiovascular system.

## Global level of A-to-I RNA editing in relation to cardiovascular disease

The advent of RNA sequencing technology has enabled transcriptome-wide systematic detection and analysis of RNA editing events. Large datasets from RNA sequencing of various types of tissue are now available, for example, such data generated by the Genotype-Tissue Expression (GTEx) project are accessible through the GTEx Portal, and have been utilized in studies of RNA editing^6^. Such studies have shown that there are millions of RNA editing sites within inverted repeated Alu elements in the human genome^36,37^. Furthermore, several hundred sites of RNA editing within protein-coding genes have been identified^38–41^.

Meanwhile, a number of RNA-sequencing data analysis methods have been developed for quantifying the global levels of RNA editing in studied cells and tissues^42^. Among such methods, the Alu editing index (AEI)^43^ is the most widely used. It determines the overall level of A-to-I RNA editing in Alu repeats, which approximates the total level of RNA editing, as most RNA editing events in human cells are A-to-I editing by ADAR1 within Alu repeat elements^43^.

Recent studies of RNA-sequencing data have revealed that there is an association between the global level of A-to-I RNA editing in cardiovascular tissues and cardiovascular diseases, as summarized below.

In a study of the global level of A-to-I RNA editing across 30,319 sites in 49 human tissues from GTEx, Li et al. found that A-to-I editing events were highly enriched at genomic loci previously identified by genome-wide association studies to be associated with CAD and various other immune-related diseases^6^. Furthermore, their study showed that the CAD risk variants were generally associated with less editing and this effect was even more significant when tested in cardiovascular tissues, indicating that reduced A-to-I RNA editing increases CAD risk^6^.

In another study, Kokot et al. compared the levels of RNA editing in biopsies from patients with heart failure due to cardiomyopathy and biopsies from patients with normal cardiac function to serve as controls. They found that A-to-I RNA editing events in 1211 genes were generally lower in patients with heart failure than in controls^44^.

However, in another analysis of the global level of A-to-I RNA editing in different tissues, Mann et al. found that patients with CAD or cardiomyopathy had a significant increase in A-to-I RNA editing in cardiovascular tissues, compared with individuals without these diseases^41^.

It is noteworthy that Li et al.^6^ and Kokot et al.^44^ each examined A-to-I in a panel of selected genes, whereas Mann et al.^41^ determined the global A-to-I editing levels.

## A-to-I editing of transcripts of individual genes in cardiovascular disease

**Fig. 2:**
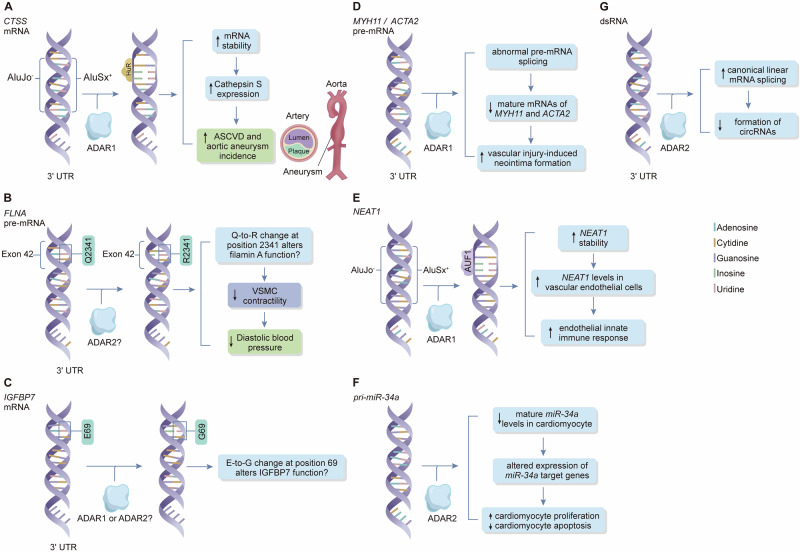
Adenosine (A)-to-inosine (I) editing of transcripts of individual genes implicated in cardiovascular disease. **A** ADAR1-mediated editing of Alu repeats in the 3’-untranslated region of the cathepsin S (*CTSS*) gene transcript facilitates its interaction with the RNA binding protein HuR, consequently increasing *CTSS* transcript stability and *CTSS* expression. Cathepsin S promotes atherosclerotic cardiovascular disease (ASCVD) and aortic aneurysm by degrading vascular extracellular matrix. **B** A-to-I editing of exon 42 of the filamin A (*FLNA*) gene transcript leads to a substitution of the glutamine (Q) at residue 2341 of the filamin A protein by an arginine (R), which reduces vascular smooth muscle cell (VSMC) contractility and therefore diastolic blood pressure. **C** A-to-I editing of the insulin-like growth factor binding protein-7 (*IGFBP7*) gene transcript results in a substitution of the glutamic acid (E) at residue 69 of the IGFBP7 protein by a glycine (G). The rate of A-to-I editing in *IGFBP7* transcripts within cardiovascular tissues is significantly elevated in patients with ischemic cardiomyopathy. **D** ADAR1-mediated editing of transcripts of the smooth muscle myosin heavy chain (*MYH11) and α-smooth muscle actin* (*ACTA2*) genes inactivates splice sites, which causes accumulation of the pre-mRNAs of *MYH11* and *ACTA2* along with a reduction of their mature mRNAs, leading to a reduction of these genes in VSMCs and consequently promoting vascular injury-induced neointima formation. **E** ADAR1-mediated editing of transcripts of the nuclear paraspeckle assembly transcript 1 (*NEAT1*) gene facilitates its binding to the RNA-binding protein AUF1, thereby increasing the stability of *NEAT1*. NEAT1 promotes endothelial innate immune response. **F** ADAR2-mediated editing of *pri-miR-34a* results in a reduction in mature *miR-34a* in cardiomyocytes. This promotes cardiomyocyte proliferation and inhibits cardiomyocyte apoptosis by altering the expression of *miR-34a* target genes including Sirt1, Cyclin D1, and Bcl2 in mice. **G** ADAR2-mediated editing of dsRNA promotes canonical linear mRNA splicing, thereby reducing the formation of circular RNAs (circRNAs). Reduction of ADAR2-mediated RNA editing increases circRNA formation in failing human hearts.

While the above investigations show an association between the global level of RNA editing and multiple cardiovascular diseases, other studies, which examine specific RNA editing sites, have found that A-to-I RNA editing in certain genes individually influences the development of cardiovascular disease (Fig. 2).

### Cathepsin S

Cathepsin S, encoded by *CTSS* gene, is a cysteine protease that plays an important role in angiogenesis and atherosclerosis^45,46^. The 3’-untranslated region of the *CTSS* transcript contains two Alu repeats which pair to form a site of ADAR1-mediated RNA editing. A study by Stellos et al. found that editing at this site promoted the binding of a protein known as HuR to this region, which increased *CTSS* RNA stability^47^. The authors showed that hypoxia, interferon-γ and tumor-necrosis-factor-α induced *CTSS* RNA editing, resulting in increased cathepsin S expression in vascular endothelial cells^47^. Furthermore, the authors found that *CTSS* RNA editing rates and *CTSS* RNA levels in human peripheral blood mononuclear cells were associated with arterial intima-media thickness, the numbers of coronary and carotid atherosclerotic plaques, and aortic aneurysms^47^.

### Filamin A

Filamin A, encoded by the *FLNA* gene, is an actin crosslinking protein that connects actin filaments and many other proteins. It plays an important role in cell structure and function^48^. The primary transcript of *FLNA* has a single, highly conserved, A-to-I editing site in exon 42^49^. A-to-I RNA editing at this site results in a glutamine-to-arginine substitution in a protein-interacting region in the C-terminus of filamin A. This enhances the ability of filamin A to interact with other cellular proteins^50^. A study by Jain et al. showed that *FLNA* A-to-I RNA editing levels in cardiovascular tissues, including the ventricle, aorta and coronary arteries, were reduced in patients with cardiovascular diseases^51^. The authors further showed that genetically modified mice lacking *FLNA* A-to-I RNA editing had increased vascular contractility, diastolic hypertension, and left ventricular wall thickness^51^.

### Insulin-like growth factor binding protein-7

Studies have shown a link between insulin-like growth factor binding protein 7 (IGFBP7) and cardiac senescence and heart failure^52,53^. A-to-I editing in *IGFBP7* transcripts in cardiovascular tissues has been reported and can result in a glutamic acid-to-glycine substitution at residue 69 of the IGFBP7 protein^38,41^. Mann et al. showed that the rate of *IGFBP7* A-to-I RNA editing in cardiovascular tissues was increased in patients with ischemic cardiomyopathy^41^.

### Smooth muscle myosin heavy chain and α-smooth muscle actin

During the development of atherosclerotic plaques and the formation of injury-induced neointima lesions, VSMCs in the arterial media dedifferentiate from a contractile phenotype to a proliferative/synthetic state, characterized by decreased expression of VSMC contractile phenotype-associated genes such as *MYH11* (which encodes smooth muscle myosin heavy chain) and *ACTA2* (which encodes α-smooth muscle actin)^54^.

A study by Fei et al. showed that ADAR1-mediated editing of *MYH11* and *ACTA2* transcripts resulted in abnormal pre-mRNA splicing and a reduction of mature mRNAs of the VSMC contractile genes *MYH11* and *ACTA2*^55^. Furthermore, the authors showed that heterozygous knockout of *Adar1* resulted in increased VSMC contractile gene expression and reduced vascular injury-induced neointima formation in a mouse model, indicating that ADAR1-mediated RNA editing influences VSMC phenotypic switching and vascular remodeling^55^.

### NEAT1

Long non-coding RNAs, defined as RNA molecules of >200 nucleotides in length that are not translated into proteins, have been found to be capable of modulating the expression of other genes and thereby influence cell behavior.

Nuclear paraspeckle assembly transcript 1 (*NEAT1*; also known as nuclear enriched abundant transcript 1) is a long non-coding RNA. The 3’ end of the *NEAT1* transcript long isoform contains 6 Alu elements. Vlachogiannis et al. detected ADAR1-mediated A-to-I RNA editing in two of these Alu elements and showed that the editing promotes binding of the RNA-binding protein AUF1 and increases *NEAT1* levels in vascular endothelial cells, and in turn increasing the expression of proinflammatory cytokines implicated in atherosclerosis, such as CXCL8^56^.

### MicroRNA-34a

MicroRNAs, defined as non-coding RNA molecules comprising 21–23 nucleotides, have been shown to modulate gene expression by targeting messenger RNA molecules.

A previous study by Boon et al. showed that silencing or genetic deletion of microRNA-34a reduced cardiomyocyte cell death and fibrosis following acute myocardial infarction in an animal model^57^. A recent study by Wu et al. identified two ADAR2-mediated A-to-I editing sites in miR-34a and showed that ADAR2 promoted cardiomyocyte proliferation and inhibited cardiomyocyte apoptosis through miR-34a RNA editing, which altered the expression of miR-34a target genes including Sirt1, Cyclin D1, and Bcl2^21^.

### Circular RNA

There is growing evidence indicating that circular RNA (circRNA), defined as non-coding RNA with a circular structure, plays a role in the development of heart failure^58^. Kokot et al. found that ADAR2-mediated A-to-I RNA editing inhibited the formation of circRNA in cardiomyocytes^44^. They reported that the levels of many circRNAs increased whilst some decreased in failing human hearts^44^.

Thus, A-to-I RNA editing of several protein-coding genes and non-coding RNA genes has been shown to influence cardiovascular cell function and has been implicated in the development of cardiovascular disease. In some cases (such as *CTSS*), RNA editing promotes cardiovascular disease, whilst in others (such as *FLNA*), RNA editing has a protective effect. Mechanistically, RNA editing can increase RNA stability, as in the cases of *CTSS* and *NEAT1*, or result in an amino acid substitution in the encoded protein, as in the case of *FLNA*.

## Detection, validation and manipulation of RNA editing

A number of methods have been reported to detect, validate, and functionally assess RNA editing events, as briefly overviewed below.

### Detection of A-to-I RNA editing sites

Global RNA editing events can been detected through analyses of whole-genome RNA-sequencing (RNA-seq) data. The particular RNA-seq methods used will have a bearing on detection of RNA editing events. For example, to detect RNA editing events in both coding and non-coding RNAs will require the construction of total RNA library for RNA-seq to capture all RNA molecules in the sample, including those without a poly(A) tail, whilst a poly(A) selection library will only captures RNA molecules with a poly(A) tail and is not suitable for studying most non-coding RNAs^59^. A total RNA library for RNA-seq can be prepared by ribosomal RNA (rRNA) depletion, as rRNA makes up the vast majority of total RNA and will mask the other types of RNA in the analysis. A strand-specific library of total RNA will allow the analysis of antisense transcripts which represent a sizable proportion of non-coding RNAs. Other factors that can impact on the sensitivity and accuracy of RNA editing detection include the sequencing depth, and the inclusion of biological and technical replicates^60^.

A number of bioinformatics tools have been developed for comprehensive detection of RNA editing events using RNA-seq data^59,61–64^. The process typically begins with aligning transcriptomic sequences to a reference genome to identify nucleotide mismatches. A major challenge is distinguishing true RNA editing events from false positives mostly arising from DNA single nucleotide polymorphisms (SNPs)^59^. To address this issue, two strategies have been reported: one by rigorously filtering out putative RNA editing sites detected in sites of known SNPs^62,65^; the other by performing paired RNA- and DNA-sequencing in the same individual and excluding sites called as SNPs and mutations from the DNA-seq data^59^. For example, it has been demonstrated that by eliminating known mouse SNP sites, RNA editing sites in the mouse brain can be predicted from RNA-seq data with high confidence^63^.

Over the years, a number of databases have emerged as repositories of known RNA editing sites that have been detected using various methods. While it is beyond the scope of this review to discuss the attributes of each database, the most recent version of REDIportal is a significant and noteworthy resource; this database contains data on ~16 million putative A-to-I editing sites^66^. Such a large collection of known editing sites is useful for scenarios where a researcher wants to quickly quantify known editing sites, saving time and computational cost of de novo detection of editing sites.

### Global quantification A-to-I RNA editing level

In addition to detecting individual editing sites, there are methods for quantifying global A-to-I RNA editing levels from RNA-seq datasets. The simplest and most straightforward method is to calculate the mean editing level of all known editing sites detected in a sample^42^. A modification of this method is to calculate mean editing level of selected sites, such as those in coding regions^41^.

As already mentioned above, the AEI^43^ is a popular metric for quantifying global A-to-I RNA editing level. The software for quantifying AEI, called RNAEditingIndexer^43^, also calculates editing index for other RNA editing types.

### Differential detection of A-to-I RNA editing

For some studies, a simple comparison of global A-to-I RNA editing between groups of samples is sufficient to indicate differential RNA editing. However, a number of tools exist for quantifying differential RNA editing between samples. Some tools like JACUSA2^67^, rnaEditr (https://bioconductor.org/packages/rnaEditr) and REDIT^68^ focus on detecting differential RNA editing events at specific sites. Other tools like LODEI^69^ and rnaEditr, focus on detecting differences between regions of the genome.

### Validation

As bioinformatic results from RNA-seq data analyses can potentially include false positive findings^70^, candidate RNA editing sites of particular interest, e.g., a specific site in the transcript of a disease-associated gene, can be validated using the following molecular experimental techniques. The presence of an RNA editing site can be confirmed by performing reverse transcription-quantitative polymerase chain reaction (RT-PCR) of a biological sample to amplify the RNA molecule under study, followed by Sanger sequencing of the amplicon or by cloning the amplicon using bacterial plasmids and then Sanger sequencing individual clones^47,71–74^. This method allows the detection of a single-nucleotide change within the RNA molecule in the sample for validation. It also provides a means of semi-quantification, which can be achieved by measuring the relative heights of the peaks of the original and new nucleotides in the Sanger sequencing chromatogram or by determining the percentage of clones with altered nucleotide(s).

An alternative method for validation of RNA editing is restriction fragment length polymorphism analysis of RT-PCR products, where the nucleotide change happens to introduce or abolish a restriction endonuclease cutting site^71^. This method also allows semi-quantitation, which can be achieved by determining the relative amounts of the cut and uncut PCR products.

### Manipulation

**Fig. 3:**
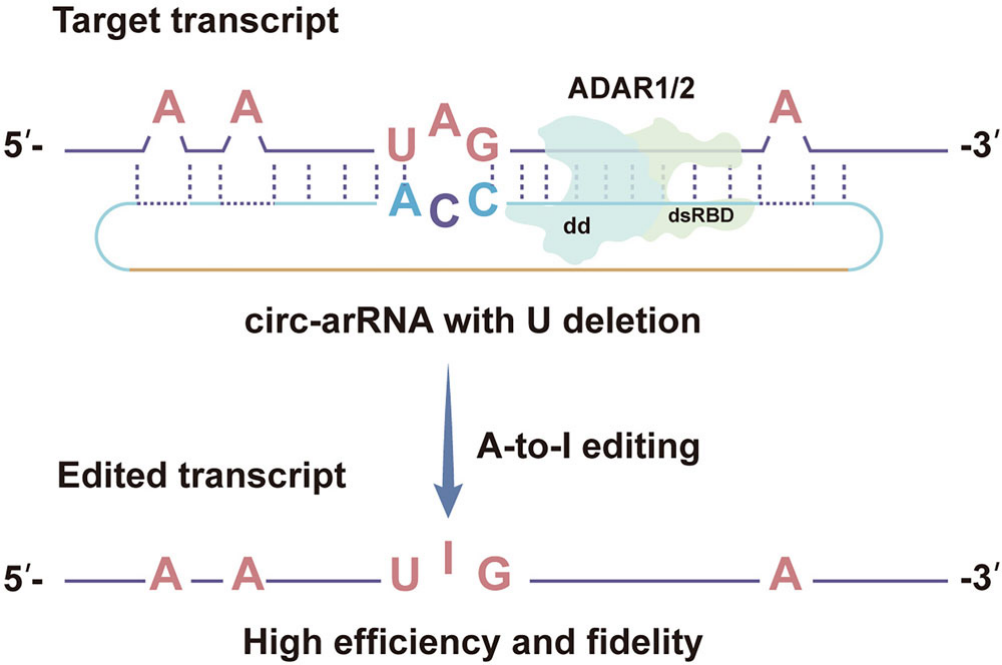
Induction of A-to-I RNA editing by LEAPER 2.0. Circ-arRNA (circular ADAR recruiting RNA) anneals with the target transcript to form dsRNA substrate, which in turn recruits endogenous ADARs for targeted editing. The abundance of arRNA in cells is positively correlated with editing efficiency. Circ-arRNA, due to its higher stability, can enhance editing efficiency. The removal of uridines opposite non-target adenosines on circ-arRNA effectively prevents off-target adenosine editing. U, uridine; dd, deaminase domain; dsRBD, dsRNA-binding domain.

Recently, site-directed RNA editing (SDRE) techniques have been developed, which are useful tools for functional characterization of specific RNA-editing events and investigation of their potential causal effects in biological or disease processes. There are comprehensive reviews of these techniques in the literature^75,76^. These techniques involve the recruitment of endogenous editing enzymes or engineered base editors to specific sites on the target RNA molecule^75,77–79^. Methods harnessing the editing capability of endogenous ADARs for A-to-I editing are relatively simpler (compared to engineered base editors) and have been shown to have high editing efficiency^79^. An example is LEAPER 2.0 (leveraging endogenous ADAR for programmable editing of RNA 2.0), which uses circular ADAR recruiting RNAs (circ-arRNAs) to recruit endogenous ADAR1 or ADAR2 to facilitate the A-to-I transition on target RNAs (Fig. 3). This method has been demonstrated to have high editing efficiency and low off-target effects in both in vitro and in vivo conditions^78^.

## RNA editing rates as a potential biomarker for cardiovascular diseases

A recent study reported that there is a strong correlation between RNA editing levels in peripheral blood cells and cardiovascular tissues^41^. Furthermore, the authors showed that the level of *IGFBP7* RNA editing in peripheral blood cells was a predictive marker for heart failure, with a predictive value comparable to that of some well-established biomarkers, such as troponin I, troponin T, atrial natriuretic peptide, and B-type natriuretic peptide^41^.

As mentioned earlier, a previous study by Stellos et al. showed that the rates of RNA editing in *CTSS* transcripts in human peripheral blood mononuclear cells were associated with arterial intima-media thickness, the number of coronary and carotid atherosclerotic plaques, and aortic aneurysms^47^, raising the possibility of using the *CTSS* editing level in peripheral blood mononuclear cells as a biomarker for atherosclerotic cardiovascular diseases.

## Engineering RNA editing as a new approach for treating cardiovascular diseases

In recent years, the possibility of using gene editing technologies to correct inborn DNA mutations for treating genetic diseases has received widespread interest^80,81^. This approach is now being tested in some patients^82^. One of the caveats of such DNA base editing is that it introduces a permanent alteration in the genome with a risk of off-target effects^83^.

In comparison, RNA base editing is reversible and has a lower risk of long-term off-target effects, as RNA base editing does not introduce a permanent change in the genome. RNA base editing only alters the sequence of the target RNA molecule(s), and RNA molecules are naturally turned over with time. Another advantage of RNA base editing over DNA base editing is that a desirable level of RNA editing can be achieved by administrating an optimized dose of the editing reagents.

Precision RNA base editing can be achieved by harnessing the endogenous ADARs or using engineered RNA base editors^75^. For example, Yi et al. demonstrated that using engineered circRNAs to recruit endogenous ADAR1 or ADAR2 to induce A-to-I editing at targeted sites of RNA molecules achieved high RNA editing efficiency both in cell culture and in a mouse model^78^. In another example, Yang et al. demonstrated that by using the CRISPR/Cas13d (CRISPR associated protein 13d) technique, it was possible to induce RNA editing to allele-specifically cleave the mutant form of β-myosin heavy chain gene transcripts, without affecting the wildtype form^84^. They showed that the application of this approach was able to prevent cardiac hypertrophy in a mouse model without apparent adverse effects, suggesting that this can be a safe and effective approach for treating hypertrophic cardiomyopathy caused by β-myosin heavy chain gene mutation^84^.

## Questions and future perspectives

In summary, there is accumulating evidence indicating that RNA editing is required for cardiovascular development and homeostasis whilst aberrant RNA editing plays an important role in cardiovascular diseases. There is a good potential that RNA editing can be leveraged in the development of new biomarkers and treatments for cardiovascular diseases.

However, there are still many remaining questions and much further research in this emerging field is required; some of these are highlighted below.

The findings that knockout of either ADAR1^29^ or ADAR2^34^ in mouse hinders the development of the cardiovascular system and that smooth muscle cell-specific knockout of ADAR1 in mouse promotes the formation of atherosclerotic lesions^20^ indicate that these enzymes play causal roles in these physiological and pathological conditions, but further studies are still required to establish causality for these and other cardiovascular phenotypes. It is likely that the deaminating activity of these enzymes is primarily responsible for such effects, but it is unclear whether some RNA editing-independent mechanisms are also involved. Both ADAR1 and ADAR2 can potentially exert RNA editing–independent effects, for example, it has been reported that these ADARs can modulate gene expression, alternate RNA splicing, and microRNA processing, independent of their RNA editing activity, and that even catalytically-inactive ADARs may have such functions^26^.

Although there is emerging evidence linking changes in the global level of RNA editing to cardiovascular diseases, it is still to be established if cardiovascular diseases are associated with an increase or a decrease in the global level of RNA editing. Furthermore, the biological mechanism underlying such an association is unknown. It is unclear if the global level of RNA editing per se has a direct pathogenic role or is only a marker for some pathogenic factors. Therefore, further studies are required to establish if global RNA editing is causative in the development of various cardiovascular diseases.

Several hundred genes have been reported to be subject to RNA editing^38–41^. So far, RNA editing in only the few genes mentioned earlier has been reported to participate in the development of cardiovascular disease; it is unclear if there are additional genes in which RNA editing contributes to cardiovascular disorders.

It is also unknown which factors and mechanisms promote or inhibit RNA editing in the development of cardiovascular diseases, and if they are preventable. Insights into such factors and mechanisms may help the development of new approaches for cardiovascular disease prevention and treatment.

A-to-I editing is the most abundant type of RNA editing, whilst C-to-U RNA editing is also highly abundant in mammals, including humans. It is well established that C-to-U editing in apolipoprotein B (apoB) transcripts in the intestine results in the production of a truncated form of the apoB protein, known as apoB-48, which is present in chylomicrons, whereas the full-length apoB protein (named apoB100) produced in the liver is present in low-density-lipoproteins^85^. Chylomicrons and low-density-lipoproteins are both atherogenic^85^. It is unclear if there are other genes where C-to-U RNA editing is associated with cardiovascular disease.

The possibility of using RNA editing level in peripheral blood cells as a biomarker warrants further investigation, as studies have shown that there is a correlation between the RNA editing levels in peripheral blood cells and those in cardiovascular tissues and there is an association between peripheral blood cell RNA editing levels in some genes (*CTSS* and *IGFBP7*) and cardiovascular diseases^41,47^. If validated, such a biomarker can be readily utilized, as peripheral blood cells are easily accessible and the measurement of RNA editing level is technically straightforward.

Engineering RNA editing as a novel approach for cardiovascular disease treatment has promising potential and warrants further development, given that it has several advantages over the DNA editing approach, as highlighted earlier. Indeed, studies are underway by several pharmaceutical companies to explore the use of RNA editing as novel treatments for a number of diseases, including cardiovascular disease^86^. As an example, ProQR Therapeutics is assessing targeting *B4GALT1* with RNA editing to introduce variants into *B4GALT1* that have been reported to improve the lipid profile in murine models of metabolic syndrome^87^ As this field is receiving considerable interest, it is anticipated that further developments of RNA editing technologies for therapeutic intervention, and assessments of their on-target specificity, efficacy, and safety in preclinical models of cardiovascular diseases, will arise in the coming years.

### Reporting summary

Further information on research design is available in the Nature Portfolio Reporting Summary linked to this article.

## Supplementary information

Reporting Summary
